# Keep calm: the intestinal barrier at the interface of peace and war

**DOI:** 10.1038/s41419-019-2086-z

**Published:** 2019-11-07

**Authors:** Lester Thoo, Mario Noti, Philippe Krebs

**Affiliations:** 1 Division of Experimental Pathology, Institute of Pathology, University of Bern, Bern, Switzerland; 2 Graduate School for Cellular and Biomedical Sciences, University of Bern, Bern, Switzerland; 3 Department of Gastro-Intestinal Health, Immunology, Nestlé Institute of Health Sciences, Nestlé Research, Lausanne, Switzerland

**Keywords:** Mechanisms of disease, Mucosal immunology

## Abstract

Epithelial barriers have to constantly cope with both harmless and harmful stimuli. The epithelial barrier therefore serves as a dynamic and not static wall to safeguard its proper physiological function while ensuring protection. This is achieved through multiple defence mechanisms involving various cell types - epithelial and non-epithelial - that work in an integrated manner to build protective barriers at mucosal sites. Damage may nevertheless occur, due to pathogens, physical insults or dysregulated immune responses, which trigger a physiologic acute or a pathologic chronic inflammatory cascade. Inflammation is often viewed as a pathological condition, particularly due to the increasing prevalence of chronic inflammatory (intestinal) diseases. However, inflammation is also necessary for wound healing. The aetiology of chronic inflammatory diseases is incompletely understood and identification of the underlying mechanisms would reveal additional therapeutic approaches. Resolution is an active host response to end ongoing inflammation but its relevance is under-appreciated. Currently, most therapies aim at dampening inflammation at damaged mucosal sites, yet these approaches do not efficiently shut down the inflammation process nor repair the epithelial barrier. Therefore, future treatment strategies should also promote the resolution phase. Yet, the task of repairing the barrier can be an arduous endeavour considering its multiple integrated layers of defence - which is advantageous for damage prevention but becomes challenging to repair at multiple levels. In this review, using the intestines as a model epithelial organ and barrier paradigm, we describe the consequences of chronic inflammation and highlight the importance of the mucosae to engage resolving processes to restore epithelial barrier integrity and function. We further discuss the contribution of pre-mRNA alternative splicing to barrier integrity and intestinal homeostasis. Following discussions on current open questions and challenges, we propose a model in which resolution of inflammation represents a key mechanism for the restoration of epithelial integrity and function.

## Facts

- The intestinal barrier is equipped with a multilayer defence system working both simultaneously and sequentially to protect against intrinsic and extrinsic noxae.
- Inflammation is essential for epithelial barrier protection but when uncontrolled, it can also damage the tissue.
- Wound healing and inflammation are inter-connected processes.
- Pre-mRNA splicing alterations are associated with intestinal pathologies.

## Open Questions

- Which molecular events or perturbations induce disequilibrium of the intestinal barrier and the establishment of chronic inflammation?

- How can we translate information from the latest microbiome studies on immune function into therapies?

- Can resolution be promoted in chronic intestinal inflammatory disorders to halt inflammation?
- Is targeting pre-mRNA alternative splicing a potential therapeutic option to promote resolution and epithelial barrier repair?

## Introduction

Epithelial organs, such as the skin, respiratory and gastrointestinal tracts, constitute a large fraction of the body that interface with the external environment (estimated surface areas of 1.7, 40 and 32 m^2^, respectively)^1–3^. Owing to their location, they encounter a variety of assaults, e.g. from pathogens, biological or chemical insults. However, in most cases the organism preserves the integrity of these barriers and thereby prevents a state of chronic inflammation.

**Fig. 1:**
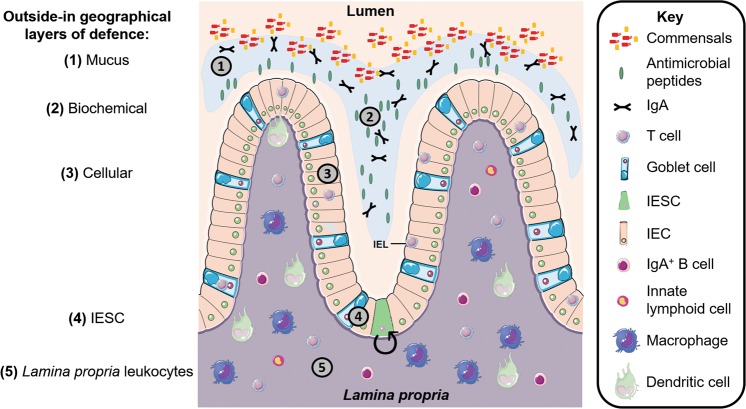
Geographical layers of intestinal (colon) defence mechanisms. The epithelial barrier consists of multiple layers of defence, which function both simultaneously and subsequently with each other. Geographically, from the outside (lumen) towards the inside (*lamina propria*): (1) the outer most layer consists of mucus which acts as a physical barrier (2) that is further reinforced biochemically with antimicrobial peptides and immunoglobulin A. (3) Intestinal epithelial cells form a single-cell layer of protection which is interspersed with intraepithelial lymphocytes. (4) Within intestinal crypts are intestinal epithelial stem cells, which are key in replenishing the epithelial surface. (5) Beyond the epithelial layer is the *lamina propria*, which is densely populated with leukocytes that serve to back up the innate immune defences and provide immunological memory against future repeated insults. Note that this graphic does not dictate the order of importance but rather serves to visualise the multiple layers of defence that make up the epithelial barrier. Abbreviations: Immunoglobulin A, IgA; intraepithelial lymphocyte, IEL; intestinal epithelial cell, IEC; intestinal epithelial stem cell, IESC. Figure adapted from stock images provided by Servier (https://smart.servier.com/smart_image/)

Besides building a physical layer, numerous epithelial- and non-epithelial cell types complement each other to form a multi-layered, highly dynamic physical, bio-chemical and immunological protection to maintain tissue homeostasis^4^ (Fig. 1 and Table 1). Importantly, this barrier system has to be selectively permeable to allow the absorption of water and nutrients, while continuously impeding harmful noxae. In most cases, the barrier remains intact, which avoids the induction of uncontrolled inflammatory responses. However, in some instances, the barrier is breached, leading to an inflammatory response to expel the invading noxae.

**Table 1:** Important players in the maintenance of the intestinal epithelial barrier at steady-state

Component	Mode of protection	References
Specialised secretory ECs		
Paneth cells	Secretion of antimicrobial peptides and factors supporting intestinal stem cells	^20^
Goblet cells	Secretion of mucins	
Sentinel Goblet cells	Specifically found at intestinal crypt entrance to protect the intestinal stem cells niche: respond to invading microbes and induce mucus secretion by neighbouring Goblet cells to expel bacteria	^79^
Mucus; consists of two dynamic layers in the large intestine, a single loose layer in the small intestine	Physical and biochemical barrier	^48,144^
Outer layer	Contains (commensal) bacteria that provide colonisation resistance, degrade nutrients for host absorption	
Inner layer	Sterile compartment: contains secreted IgA, antimicrobial peptides	
Secretory immunoglobulin A (sIgA)	Natural IgA provide immune exclusion of microbes from the epithelium and prevents over-stimulation of the mucosal immune system	^49,145^
	Induction is dependent on microbes	
	Commensal-complexed sIgA reduce inflammatory cytokine levels (IL-8, TNF, IL-1β)	^146^
	High-avidity pathogen-specific IgA: clusters fast replicating bacteria for subsequent clearance by the natural peristaltic flow of intestinal contents	^147^
	Prevents interaction with IECs and unnecessary inflammation	
Antimicrobial peptides	Directly kill or inhibit microbial growth	^148^
Immune cells	Immunity against pathogens	
Dendritic cells (DCs)	Found in the *lamina propria* below the epithelium	
	Sample for luminal antigens via transepithelial dendrites	^19^
	Promote intestinal repair	^46^
Intraepithelial lymphocytes	Located in the epithelium	^149^
TCRγδ+	Secrete factors (e.g. TGFβ1, TGFβ2, KGF) to support & maintain the epithelial barrier	
TCRαβ+	Have cytotoxic activity	
Innate lymphoid cells	Found in the *lamina propria* below the epithelium	^95^
	Action via IL-22 which promotes intestinal tissue repair, protects from intestinal pathogens and restricts particular microbiota	
Macrophages	Sample luminal content, engulfment of invading bacteria and apoptotic cells and maintain epithelial integrity	^150^
Commensal microbiota	Provide colonisation resistance	^151^
	Break down complex diet molecules for host uptake	
	Bacterial-derived stimuli from the luminal-side provide signals for the epithelial barrier maintenance	^30,92^
	Educate the mucosal immune system	^152^

*DCs* dendritic cells, *ECs* epithelial cells, *IgA* immunoglobulin A, *sIgA* secretory IgA, *TNF* tumour necrosis factor, *TGF* transforming growth factor, *KGF* keratinocyte growth factor, *TCR* T cell receptor, *IL* Interleukin

**Fig. 2:**
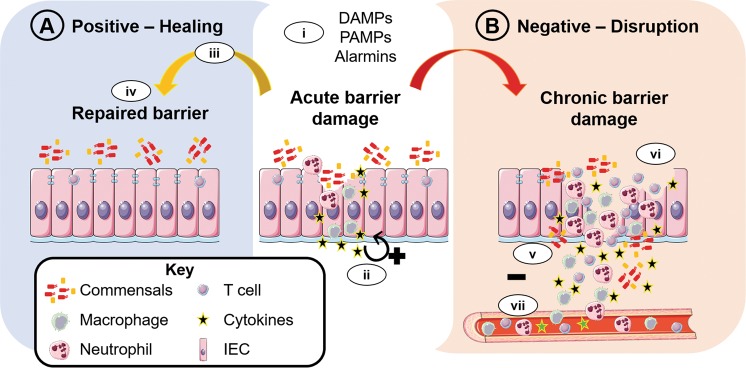
Damaging and healing properties of inflammation at barrier sites. **a** Acute barrier damage induces an inflammatory response, which starts as a localised response to help repair the barrier: (i) Damage and release of alarmins (e.g. IL-33) and (ii) localised inflammatory cytokine release (e.g. IL-6 and TNF) activate tissue myeloid cells to clear harmful noxae and promote IEC proliferation; (iii) the inflammation phase is shadowed by a resolution phase (iv) which successfully shuts down inflammation and permits the restoration of the barrier. **b** Chronic inflammation induces further barrier damage: (v) If inflammation becomes uncontrolled, this creates a pro-inflammatory microenvironment due to the increased cytokine release and leukocyte infiltration, (vi) increased barrier disruption occurs due to the actions of pro-inflammatory leukocytes leading to (vii) systemic involvement of the immune system and chronic inflammation at the barrier. Abbreviations: Intestinal epithelial cell, IEC; damage-associated molecular patterns, DAMPs; pathogen-associated molecular patterns, PAMPs; interleukin-33, IL-33; interleukin-6, IL-6; tumour necrosis factor, TNF. Figure adapted from stock images provided by Servier (https://smart.servier.com/smart_image/)

The multiple and redundant lines of defence that have developed during evolution to maintain the barrier highlights the selective pressure of investing energy to prevent disruption of the barrier in the first place. This strategy of prevention, instead of constantly mounting an inflammatory response to expel the insult, is energetically economical for the host^5–7^. Indeed, although the inflammation process commonly leads to the clearance of the harmful noxae, tissue damage can also occur from persistent or uncontrolled inflammation, requiring the host to expend further energy to repair and restore barrier integrity and function. Inflammation is a complex process affecting not just the immune system but also physiological processes such as induction of the acute-phase response and fever, thus affecting multiple organs and functions^8^. Initially, the inflammatory response acts locally to eliminate the insulting agent and restore barrier function (Fig. 2a). However, high noxae load and sustained barrier damage may also activate systemic inflammatory responses (Fig. 2b). An initial localised response to the noxae instead of a systemic reaction is more beneficial both at a metabolic energy level but also to prevent unnecessary systemic inflammation that is accompanied by fever, pain, anorexia and somnolence^8^.

In this review, we focus on the intestines as a model epithelial barrier to highlight the importance of barrier integrity for host fitness. We discuss how inflammation affects the barrier on multiple levels, and stress the importance of resolution (i.e. the active host mechanism to terminate inflammation)^8–10^. While the complexity of a multi-layered protection system makes the barrier more resistant to damage and infection, restoring barrier integrity and function in the context of chronic inflammation proves to be a challenging task.

Since intestinal barrier integrity is critically dependent on intestinal epithelial cell (IEC) fitness, mechanisms affecting IEC function are important parameters that regulate the epithelial barrier. Evidence for the essential relevance of the epithelial barrier is further supported by animal models in which targeted deletion of key IEC components increased susceptibility to colitis development^11–13^. Since proteins are the means for a cell to carry out its functions, upstream processes such as post-transcriptional modifications can alter cell function. For instance, pre-mRNA alternative splicing (AS) generates a variety of proteins from the same transcript, resulting in proteins of complementary or even opposing functions^14^. Indeed, dysregulation of AS has recently been linked to barrier defects^15,16^. We therefore discuss the impact of AS for intestinal health and pathology. Finally, we discuss combined approaches to target inflammation, resolution and barrier repair to treat chronic intestinal inflammation.

## The Barrier’s Toll: detecting harmful noxae

Intestinal commensals exist in symbiosis with the host, with both benefiting from the metabolic energy sources provided reciprocally^17,18^. However, when barrier integrity is compromised (Fig. 2), intestinal microbes (both opportunistic commensals and pathogens) and microbial-derived products have direct access to the inner mucosa and blood vessels, posing a risk for systemic infections. A key step in containing the infection and mitigating systemic dissemination is the induction of inflammation to eliminate the damaging insult, resulting in clearance and neutralisation of harmful noxae along with subsequent barrier repair. ECs and innate immune cells (such as tissue-resident macrophages and dendritic cells) sense microbial signals both directly from the microbes and factors released by infected cells (pathogen-associated molecular patterns; PAMPs) via evolutionarily conserved pattern recognition receptors (PRRs), thereby initiating an inflammatory cascade inducing the secretion of cytokines and chemokines for the recruitment of myeloid immune cells^19^.

IECs are among the first responders to microbes and express a wide range of PRRs ranging from extracellular and endosomal membrane-bound Toll-like receptors (TLRs) to cytoplasmic RIG-I-like receptors (RLRs) and NOD-like receptors (NLRs)^20^, which enable the detection of microbial molecules. Yet, ligation of PRRs on IECs do not always result in inflammation activation - there is a selective inhibition or initiation of inflammation depending on whether the PRR-stimuli originates from their apical or basolateral sides, respectively^21,22^. In particular, TLR5 is specifically expressed on the basolateral side of IECs, permitting responses to bacterial flagellin of invasive, epithelial-translocating bacteria (e.g. *Salmonella*) but not commensal *Escherichia coli*, which does not translocate^21^. Furthermore, in vivo rectal administration of flagellin into mice with injured colonic mucosa but not intact mucosa led to mitogen-activated protein kinase 1/2 (MEK1/2) activation downstream of TLR5 signalling, indicating that commensal-derived flagellin can serve as pro-inflammatory stimuli in injured intestines^22^. This anatomical segregation of inflammation signalling is advantageous to prevent uncontrolled inflammation against commensal microbes, while permitting inflammation only when microbes infiltrate into the sterile compartments, indicative of damage in the epithelial barrier and the need to repair it.

Inflammation can also be induced by non-microbial stimuli, such as sterile cellular damage (i.e. transformed cells, physical damage, UV-irradiation on the skin), which eventually initiates wound repair^23^. Damage-associated molecular patterns (DAMPs), similarly to PAMPs, possess conserved molecular patterns recognised by PRRs expressed by ECs and several other cell types at mucosal sites. DAMPs are often intracellular components (e.g. nucleic acids, ATP) which are released by damaged or necrotic cells^23^. Alarmins, which include interleukin (IL)-33 and IL-1α, are a subset of DAMPs with chemotactic and activating functions on immune cells to clear damaged or necrotic cells or amplify immune function^23,24^. How a cell dies influences the inflammatory responses - controlled apoptotic death is self-containing and typically less inflammatory, unless apoptotic cell clearance is impaired.

Asides from released intracellular DAMPs, damaged or transformed ECs also upregulate cell surface stress ligands such as retinoic acid early inducible-1 (RAE-1; in mice) or MHC class I-related protein A (MICA; in humans)^25,26^. Upregulation of these stress ligands activate natural killer (NK) receptors such as natural killer group 2D (NKG2D) expressed on intraepithelial lymphocytes (IELs). Activated IELs then kill stressed ECs and release pro-inflammatory cytokines, such as tumour necrosis factor (TNF) and interferon (IFN)γ^27^. Considering that microbial stimuli and epithelial damages are themselves able to induce inflammation, it is essential that both the damaging noxae is cleared and that the barrier is repaired to prevent the establishment of a chronic inflammatory condition.

## Commensal stimuli promote barrier repair

Although microbial stimuli are best known to induce intestinal inflammation, commensal-derived products (including metabolites) and PRR-signalling also maintain and facilitate repair of the epithelial barrier^28,29^. In support of PRR-signalling importance for both sensing noxae and to initiate barrier repair, mice deficient in myeloid differentiation primary response 88 (*Myd88*), which encodes a key downstream adaptor protein of all TLR [except TLR3 that signals exclusively via TIR-domain-containing adapter-inducing interferon-β (TRIF/TICAM-1)], are susceptible to experimental colitis induced by dextran sodium sulfate (DSS), a chemical that damages the colonic epithelium^30^. This was in part due to the reduced proliferation of the IECs following DSS-triggered damage thereby diminishing barrier repair^30^. Defective MyD88-signalling specifically in IECs reduced host survival in the *Helicobacter hepaticus*-induced model of colitis, suggesting a key role of TLR-sensing by IECs for barrier restoration^31^.

Since IECs express a range of TLRs, albeit at lower expression levels than leukocytes^32–39^, it is conceivable that TLR-signalling in IECs is directly responsible for initiating the above-mentioned damage-induced secretion of IL-6, TNF and CXCL1 to promote IEC repair. Further underlying the importance of IEC-specific TLR-sensing, the MyD88-downstream activation of nuclear factor kappa-light-chain-enhancer of activated B cells (NF-κB) maintains IEC proliferation and survival^40,41^. Defective NF-κB signalling in IECs due to deletion of NF-κB essential modulator (NEMO) increases TNF-induced apoptosis, thus resulting in spontaneous chronic intestinal inflammation in mice^42^. Supporting the need to regulate TNF-induced death in the intestines, IECs express Caspase-8 to protect them from TNF-induced death and to regulate their turnover rate, as exemplified by the development of ileitis in IEC-specific Caspase-8 deficient mice^43^.

Asides from IEC-mediated TLR-sensing, NF-κB signalling is also important for immune function, also in the intestine^44,45^. Particularly, in the DSS-colitis model, MyD88-signalling in B cells (and to a lesser extent in CD11c^+^ dendritic cells) is critical to promote intestinal repair^46^. However, in the *H. hepaticus*-colitis model, MyD88-activation within innate cells leads to worse intestinal inflammation^31^. However, this effect is likely specific to this pathogen as MyD88-signalling is protective during infection with other intestinal pathogens, including *Salmonella* and *Citrobacter rodentium*^47^.

The barrier repair’s dependency on immune cells is likely related to the need to both control commensal outgrowth at the damaged site and to provide reinforced protective factors, such as immunoglobulin A (IgA) to the barrier defences to prevent further microbial invasion^48,49^ (Fig. 1). Important growth factors for ECs such as epidermal growth factor (EGF) and amphiregulin (AREG) are also secreted by resident or infiltrating immune cells^50^.

Commensals play a significant role in developing the mucosal immune system, influencing the intestinal barrier’s homeostatic defences and turnover rate, intestinal inflammation and pathologies, which has been the topic of several recent reviews^51–53^.

## Inflammation alters the epithelial barrier

Once the epithelial barrier has been breached by pathogens or by physical or chemical insult, PAMPs/DAMPs activate IECs to secrete chemokines (e.g. chemokine ligand 20; CCL20), and tissue-resident myeloid cells to secrete lipid-derived mediators (e.g. prostaglandins and leukotrienes). CCL20 gradients attract chemokine receptor 6 (CCR6)-expressing immune cells including dendritic cells, neutrophils and macrophages, which survey the epithelium for noxae^54^. Additionally, lipid-derived mediators are potent chemo-attractants of neutrophils^55,56^ which are recruited to clear harmful insults. During the inflammatory process, infiltrating neutrophils migrate through the epithelium, temporarily disrupting the epithelial barrier by breaking IEC intercellular junctions^56^. This barrier disruption has been shown in the lungs to provide stimuli for increased EC proliferation via β-catenin signalling, and thereby promote later barrier repair^57^. Such integrated response of barrier disruption to allow neutrophil transmigration while simultaneous signalling to increase barrier repair exemplifies the dynamic nature of the epithelial barrier and the relevance of a prompt restoration of the barrier.

Within the tissue, neutrophils exert their characteristic functions of phagocytosis, neutrophil extracellular trap formation, and the degranulation of antimicrobial proteins, reactive oxygen species and cytokines to ensnare and eliminate the harmful noxae^58^. Cytokines and chemokines secreted by both IECs and myeloid immune cells within the damaged site help create an inflammatory milieu conducive to the clearance of the damaging noxae. Cytokines secreted during intestinal damage such as IL-6, TNF and chemokine C-X-C motif ligand 1 (CXCL1, also known as KC-1) have dual roles on different cell types (Fig. 2): promoting tissue repair by the regulation of IEC proliferation, yet also acting as pro-inflammatory factors on immune cells^30^. These inflammatory mediators act locally to further activate macrophages and neutrophils in the damaged tissue, but they may also induce the acute-phase response in the liver, and the subsequent symptoms of fever and fatigue if produced in larger quantities^8^.

## Inflammation’s detrimental impact on different cell types in the intestinal epithelium

### *Loss of barrier integrity through impairment of inter-cellular interactions*

The local inflamed intestinal microenvironment consisting of recruited leukocytes and high local concentrations of cytokines have beneficial noxae-clearing and support IEC proliferation in epithelial barriers but may also contribute to a leaky barrier when excessive. Inflammatory cytokines such as TNF and IFNγ can disrupt the epithelial barrier by downregulating tight junctions (claudin-1, occludin, zonula occludens protein-1)^59^ and adherens junctions (E-cadherin) in IECs^63^, thereby compromising the physical barrier, one of the key “layers of defence” (Fig. 1). This in turn reduces the epithelium tightness and impairs the architecture of the intestinal crypt, particularly in the colon^60^. Such increased susceptibility of the colonic crypt may be related to the reduced cell-cycling rate of the intestinal epithelial stem cells (IESCs) present in the colon compared to the small intestines^61^. This may explain why the colon shows reduced capability to replenish its crypts. Besides this impact on the epithelial tightness and architecture, E-cadherin loss in IECs also compromises the maturation and positioning of goblet cells and Paneth cells, further impairing mucus production and increasing susceptibility to bacterial infection^60^. Paneth cells not only secrete antimicrobial peptides, but also various growth factors supporting and regulating IESCs in the intestines^62^. These include EGF, transforming growth factor α (TGFα), Wnt family member 3 (Wnt3) and Notch-ligand delta like 4 (Dll4)^63^. As the IESC niche is a source for EC replenishment of the epithelial barrier (and thereby serves as one of the deeper layers of defence by virtue of its epithelial maintenance function; Fig. 1), excessive inflammation-induced damage to the IESC niche can severely impair function and architecture. Indeed, colon shortening is macroscopically observed in highly inflamed mouse intestines^64^ and narrowing in chronically inflamed human intestines^65^.

#### Impact of inflammatory cues on IEC function via perturbation of intracellular processes including pre-mRNA AS

Inflammation not only impacts inter-cellular connectivity (e.g. downregulation of tight junctions) but also promotes intracellular changes such as DNA methylation and post-transcriptional modifications^66–68^. In particular, alterations of mRNA splicing has been described in inflammatory bowel disease (IBD), both within IECs^69^ and leukocytes^70^. The main regulator of AS in ECs is the epithelial splicing regulatory protein 1 (ESRP1) which maintains the epithelial identity of a cell and regulates epithelial-to-mesenchymal transition^15,71,72^. ESRP1 is conserved across species^16^ and deletion of *Esrp1* in mice results in lethal morphological defects in the skin and craniofacial malformation^15^.

In support of ESRP1-mediated AS’s role in epithelial barrier integrity, we recently showed that the intestinal barrier integrity of mice with dysregulated *Esrp1* function is compromised^16^. Specifically, mutant mice with altered ESRP1 function show reduced barrier integrity and translocation of commensals to the mucosa, resulting in increased susceptibility to DSS-colitis. This phenotype in *Esrp1* mutants was mediated by reduced proliferative capacity of IECs^16^. Additionally, we found that inflamed biopsies of IBD patients have lower *ESRP1* transcript levels compared to matched non-inflamed tissue^16^. In corroboration with this, mice with double knock-outs of *Esrp1* and its paralog *Esrp2* in the epidermis have defective tight junction proteins^73^. Based on these data, we propose that AS controls intestinal barrier integrity via modulation of tight junction proteins and regulation of proliferative capacity of IECs, which if dysregulated predisposes the host to chronic inflammation and associated tissue damage.

#### Role of microbe-derived cues on IECs as an addition to host-released inflammatory molecules

In the intestines, commensal bacteria ferment dietary fibres into short-chain fatty acid (SCFA) metabolites such as butyrate, acetate and propionate^74^ which are recognised directly by IECs via receptors such as GPR41/GPR43^75^. SCFAs serve as important energy sources of ATP for colonocyte function^76,77^. However, in chronic inflammation, with increasing epithelial damage and erosion of the crypts, butyrate can more easily reach the IESC niche. Although butyrate provides IECs with energy, it has inhibitory effects on IESC proliferation and EC replenishment^78^. During homeostasis, differentiated colonocytes (positioned further away from the crypts) metabolise butyrate, thereby decreasing its concentration towards the crypts and preventing the inhibition of IESC proliferation^78^. This exclusion of the inhibitory butyrate from IESCs is particularly relevant in the context of epithelial repair when there is a need for IESC proliferation at an increased rate. As the IESC niche plays an important role in epithelial maintenance, this site is protected by specialised sentinel goblet cells (SenGCs) located at the colonic crypts’ entrances^79^. SenGCs trigger neighbouring goblet cells to increase mucus production following TLR-activation in an effort to control microbial infiltration^79^. However, since inflammation can compromise goblet cell maturation and positioning^60^, this mechanism is compromised in protecting the IESC during inflammation.

Altogether, inflammatory damage in the epithelial surface may reach the IESC niche and thereby lead to a chain-reaction of barrier impairments and chronic inflammation in the intestines. While the integrated protective mechanisms of the intestinal barrier are advantageous to prevent (mild) damage and infection in the first place, a multi-hit disruption on multiple layers of defence (Fig. 1) - e.g. as it occurs during chronic inflammation (Fig. 2b) - makes it challenging to re-establish homeostatic balance.

## Inflammation-induced microbial dysbiosis

Inflammation-induced intestinal barrier damage often perturbs the symbiotic relationship between commensals and the host. Inflammation alters the intestine’s oxidative and metabolomic environment - factors which the commensals are dependent upon for their survival and growth^80^. This generally involves a deviation of the commensal population from a healthy, diverse symbiotic profile into a flora with typically reduced complexity and over-representation of particular taxa of microbes^77,81^. Perturbations to the structure of commensal microbial communities, referred to as dysbiosis, is frequently observed in intestinal immunopathologies such as IBD^81–83^ but also in other diseases with barrier dysregulation such as cancer^84,85^, allergies^86^, obesity^87^ and graft-vs-host disease^88,89^.

Commensals contribute to the overall intestinal barrier maintenance via their fermentation products such as SCFAs, which act as stimuli for IECs^90–92^. In line with this, IBD patients with a compromised intestinal barrier integrity have alterations in SCFA-producing bacteria^83^. It is therefore conceivable that inflammation-mediated dysbiosis can further compromise the barrier integrity as the crosstalk between commensals, IECs and mucosa-associated immune cells becomes dysregulated^55^.

The conundrum of whether dysbiosis precedes inflammation or vice versa is that it is likely that they are interdependent events. Studies have shown that inflammation alters the intestinal environment thereby reshaping microbial populations, yet gnotobiotic animal studies have also implicated that dysbiosis can predispose animals towards intestinal inflammation. This quasi philosophical question is further elaborated on in a review by Ni et al. which summarises IBD and dysbiosis associations^80^.

## Inflammation and excessive (adaptive) immune activation

Apart from alterations to IECs and the commensal community structures, barrier damage can also alter yet another layer of defence, the mucosal immune system (Fig. 1). The mucosal immune system, the commensal microbiome and the IECs form a tripartite network in the intestines - interacting with each other for growth factors and signals beneficial for their development^93–95^. The intestine is the largest immunological organ harbouring many tissue-resident immune cells throughout its length^96,97^.

In the context of uncontrolled or chronic inflammation, the pro-inflammatory microenvironment and the damaged barrier perpetuate constant immunological activation and recruitment of immune cells through sustained exposure to microbial signals. Inflamed tissues of IBD patients show increased infiltration of leukocytes from both the innate and adaptive immune system (Fig. 2b)^98^. IBD is a heterogeneous disease, in the inflamed mucosa of Crohn’s disease (CD) patients, inflammation is most commonly driven by pathological T-helper cells 1 (Th)1/Th17 responses and their associated cytokines, IFNγ and IL-17 respectively. However, in ulcerative colitis (UC) Th2 cells and their signature cytokines IL-4 and IL-13 predominate^99^. As Th1/Th2/Th17-derived cytokines have been shown to inhibit the stem cell renewal of IESCs and their direct differentiation into Paneth cells (Th1/IFNγ-induced) or Tuft cells (Th2/IL-13-induced)^100^, it is conceivable that these cytokines further impair epithelial repair during IBD. In contrast, IL-10 from regulatory T cells (Tregs) promote stem cell renewal^100^.

## Post-inflammatory healing: the restorative side of inflammation

Damage and loss of epithelial architecture are unwanted side-effects of chronic inflammation. However, in most instances, inflammation is self-limiting and is overall beneficial for the host in preventing infections, as suggested by the evolutionarily conservation of multiple pathways of inflammation in different taxonomic clades^101–103^. Inflammation is critical for adapting to intrinsic and extrinsic challenges. Yet, for inflammation to be beneficial, it has to be properly regulated.

## Resolution shadows inflammation for a balanced and beneficial host response

The role of resolution (=restoration to homeostasis) is a rather neglected aspect of inflammation^10^ that is distinct from immunosuppression (=dampening inflammation-sustaining events). Despite inflammation onset being recognised to be an active and controlled process, the resolution phase has mistakenly been assumed to be a passive process in which inflammation simply wanes.

Rather, simultaneously with the active down-regulation of inflammation, resolution is actively promoted by specialised pro-resolving lipid mediators (SPMs) which include maresins, resolvins, protectins and lipoxins, produced as a result of enzymatic cleavage of omega-3 (ω-3) and ω-6 dietary essential polyunsaturated fatty acids (PUFA)^104^. Endogenously, ω-3 and ω-6 PUFA are incorporated into the cellular membranes of all tissues where they can be utilised for transformation into pro-inflammatory (e.g. prostaglandins and leukotrienes) lipids or pro-resolution SPMs^105,106^.

SPMs have short half-lives and act in an autacoid manner^104^. They are secreted by many cell types found in the inflammatory environment (neutrophils, monocytes, macrophages, innate lymphoid cells, ECs and platelets) therefore providing a spatio-temporal control of inflammation^104,107^. Appropriate spatio-temporal synthesis and action of SPMs is key to balancing the benefits of inflammation for clearance of harmful antigen and the prevention of tissue damage.

As inflammation progresses and damage signals decrease, progressive “class-switching” of lipid mediators from the pro-inflammatory prostaglandins and leukotrienes into pro-resolving lipoxins occur^108^. SPMs subsequently halt neutrophils from infiltrating into the inflamed site as the damage signal is cleared^109^ while promoting the survival of IECs^110,111^, the production of antimicrobial peptides by IECs^110,112^, increased phagocytosis of bacteria and apoptotic cells by macrophages^113^, efferocytosis (macrophage-mediated clearance of apoptotic neutrophils which came into the damaged site to clear the harmful antigens)^114,115^, and the secretion of anti-inflammatory IL-10^114^. These concerted effects serve to enable barrier restoration and critically act beyond the initiating events of inflammation.

**Fig. 3:**
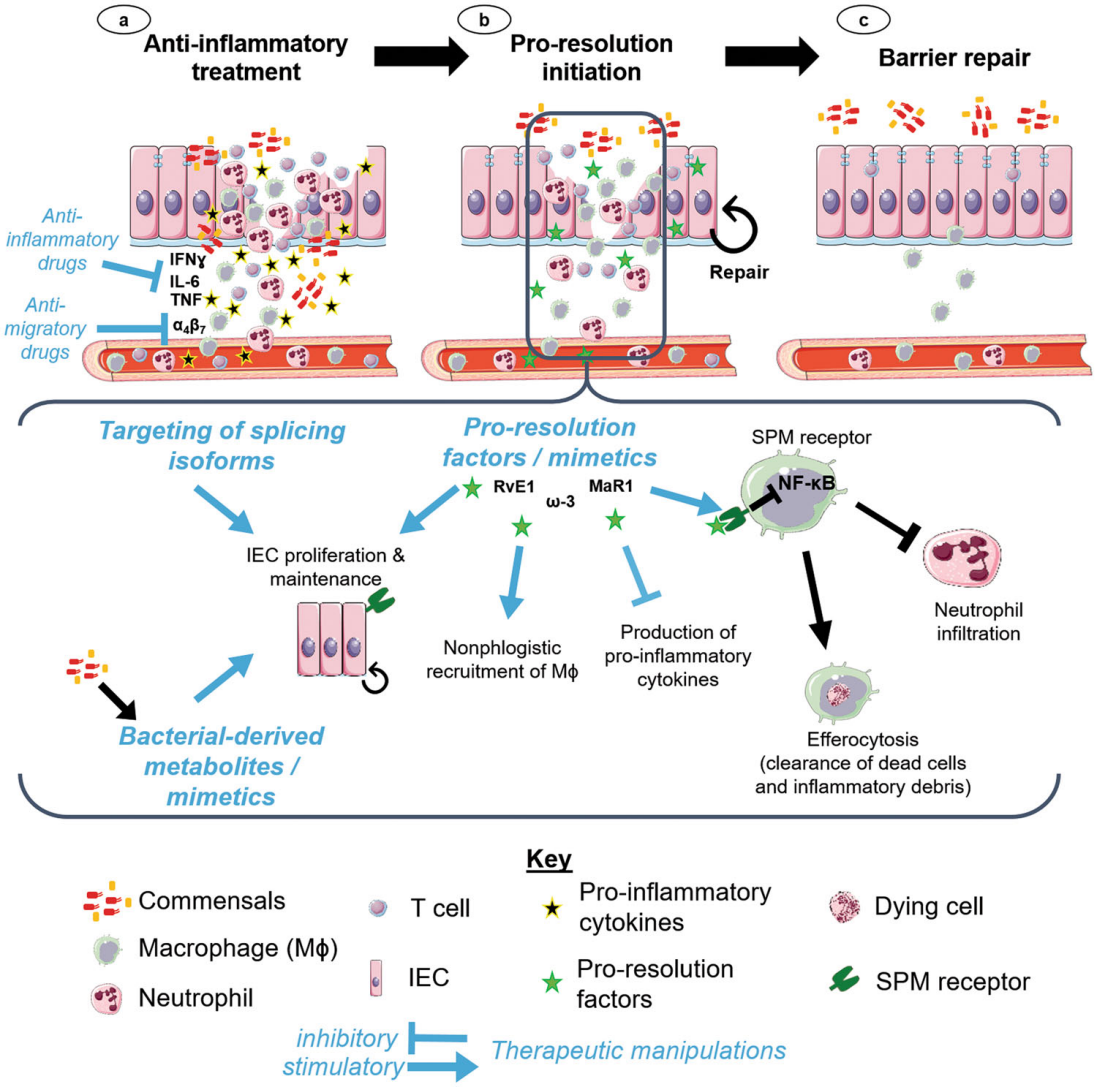
Combining strategies to target inflammation, resolution and epithelial barrier repair. **a** Dampening the inflammatory response in the damaged barrier is critical to allow resolution mechanisms to take place. Current therapeutics for intestinal inflammation (e.g. IBD) utilise anti-inflammatory and/or anti-migratory drugs. **b** The resolution phase involves conversion of pro-inflammatory lipid mediators such as leukotrienes and prostaglandins into specialised pro-resolution mediators such as resolvins. In addition, other cytokines such as IL-22 and IL-10 help to further dampen inflammatory responses while IL-33 and growth factors such as EGF promote IEC repair. Future therapeutic interventions may foster resolution by using pro-resolving factors or synthesised mimetics. Promotion of IEC repair and maintenance could also be enhanced by targeting specific splicing isoforms or via the application of bacterial-derived metabolites as their specific cellular targets and mode of action become better delineated (**c**). Combination of anti-inflammatory treatments with therapeutic promotion of resolution and epithelial barrier repair restores a functional barrier to prevent further inflammation. Areas for therapeutic manipulations are indicated by blue text and arrows. Abbreviation: alpha-4 beta-7 integrin, α_4_β_7_; epidermal growth factor, EGF; interferon γ, IFNγ; interleukin 6, IL-6; interleukin 10, IL-10; interleukin 22, IL-22; interleukin 33, IL-33; intestinal epithelial cell, IEC; macrophage, Mφ; maresin 1, MaR1; nuclear factor kappa-light-chain-enhancer of activated B cells, NF-κB; omega-3, ω-3; resolvin E1, RvE1; specialised pro-resolving lipid mediators, SPM; tumour necrosis factor, TNF. Figure adapted from stock images provided by Servier (https://smart.servier.com/smart_image/)

While SPMs are important players in initiating resolution, other mechanisms work synchronously to repair the barrier. IL-33 and its receptor ST2 are up-regulated immediately following DSS-colitis and during the intestinal barrier recovery phase, which promotes intestinal wound healing in mice^116^. Other cytokines which limit intestinal inflammation and maintain tissue homeostasis are IL-10 and IL-22, both members of the IL-10 cytokine family^95,117^. Furthermore, direct effects of growth factors such as EGF and transforming growth factor β (TGFβ) on IECs for increased proliferation^9^ and increased mucus production from goblet cells^118^ act to both replenish and reinforce the barrier (Fig. 3).

Through the orchestrated effort of halting inflammation and turning on wound healing processes, SPMs along with other resolution mediators promote the intestinal barrier’s recovery and the return to tissue homeostasis.

## Not all repairs are successful

Barrier repair is clearly beneficial to stop the uncontrolled infiltration of harmful noxae. However, repair of the intestinal barrier can vastly differ in its success following self-limited inflammation versus dysregulated chronic inflammation. In the best-case scenario of self-limited inflammation, the following steps occur for timely barrier restoration: (1) influx of neutrophils with localised action, (2) harmful antigen clearance, (3) resolution activation, (4) clearance of infiltrating neutrophils and pro-inflammatory milieu by macrophages, (5) macrophage death and clearance, (6) wound repair (see also Fig. 3).

However, in scenarios of chronic inflammation, several of these steps are compromised. A side-effect of tissue repair in a chronically inflamed tissue is the development of fibrosis and scarring^119^, which can impair normal tissue function due to the loss of elasticity and healthy structure. Intestinal fibrosis develops due to the excessive production of extracellular matrix (ECM) by activated mesenchymal cells leading to luminal narrowing. This is one of the main indication for surgery in CD patients, and post-surgery disease recurrence is common^120^. While various inflammatory cytokines such as TGFβ and IL-13 promote intestinal fibrosis, it is not certain if there are also inflammation-independent mechanisms that trigger fibrogenesis^121^. In particular, anti-inflammatory drugs only marginally impact on fibrosis^121^. ESRP1-mediated AS may also play a role in fibrogenesis by modulating epithelial-to-mesenchymal transition (EMT), a process that occurs during embryonic development but is also important for wound healing, fibrosis and cancer progression^122^. In vitro silencing of *ESRP1/2* led to a mesenchymal-like splicing signature, cellular morphology and motility thereby establishing the basis for generating repair/fibrosis-associated mesenchymal cells^71,72^.

In addition to tissue fibrosis and scarring resulting from continuous damage occurring in the inflamed tissue, cell death becomes an additional inflammatory stimulus. Although apoptosis is a programmed cell-death process, in the case where increased apoptosis rate is not balanced by a corresponding higher clearance of apoptotic bodies, secondary necrosis of these apoptotic bodies may occur which releases potentially toxic intracellular contents into the inflamed milieu^123^.

Proper timing of pro-resolving SPM synthesis and action is critical to enable both an effective inflammatory response to occur while preventing excessive tissue damage. As SPMs are produced by transcellular biosynthesis and are rapidly degraded in the local environment by myeloid cells^124^, chronic inflammatory conditions speed up the degradation of SPMs. Therefore, while the rapid degradation of SPMs by myeloid cells is beneficial to allow an inflammatory response to occur, the instability of SPMs becomes an issue in conditions of uncontrolled inflammation.

## Negotiating peace at the intestinal barrier: current therapeutics and outlook

In the context of infections, inflammation resolution occurs following the clearance of the pathogen. However, in chronic inflammatory diseases such as IBD, with undefined aetiology, it is difficult to determine the nature of the initiating damaging insult, and therefore how to clear it - both from the viewpoint of the immune system and for therapeutic intervention. Current knowledge on IBD indicates there are genetic contributions such as nucleotide-binding oligomerization domain-containing protein 2 (NOD2) mutations which affect downstream NF-κB signalling (key protein complex for immune cell activation) in myeloid immune cells^125^ or autophagy related 16 like 1 (ATG16L1) mutations, which alter the autophagosome pathway used to process intracellular pathogens^126^. Genome-wide association studies (GWAS) have additionally highlighted over 160 genetic risk loci for IBD^127^ although the majority of the loci are in non-protein coding regions of the genome^128^ and cluster within the gene regulatory elements in both IECs and immune cells^129^. However, environmental factors are also strong contributors as IBD prevalence is highest in the Western world. However, the current greatest increase in IBD incidence is occurring in newly industrialised countries^130^. Despite its multi-factorial aetiology, a characteristic of IBD is the disruption of the epithelial barrier, which allows unrestricted interaction of the commensal microbes with the IECs and the immune cells and therefore contributes to the sustained mucosal inflammation^131^. Repairing the barrier in IBD is therefore important to prevent damage from spreading faster than repair can occur.

As IBD is an immune-mediated disease, the focus of many therapies for IBD have been on dampening inflammation^132^. Current front-line therapy for IBD is the use of general anti-inflammatory and immunomodulatory drugs (Fig. 3a) including steroids, antibodies against cytokines (e.g. anti-TNF, anti-IL-12, anti-IL-23) and thioguanine nucleotides which suppress T cell responses^9,133^. However, only 50% of treated patients respond to these drugs^134^. More localised therapies include targeting leukocyte-expressed integrins (e.g. using anti-β_7_ and anti-α_4_β_7_ antibodies) which bind to the mucosal addressin cellular adhesion molecule-1 (MAdCAM-1) within the intestines, thereby reducing inflammation^135^. The use of these anti-integrin antibodies have a very strong safety record and are particularly suited for IBD patients in remission^135^. Despite these therapeutic options, many patients still fail to respond, eventually lose response over time or develop antibodies against the drugs^136^. Janus kinase (JAK) inhibitors, which work by disrupting the JAK-signal transducers and activators of transcription (STAT) signalling pathway downstream of many cytokine receptors, have recently been suggested for second-line treatment of moderate to severely active UC, as they improved outcomes by inducing remission and mucosal healing^136^.

While these therapies mostly aim to dampen inflammation, we propose that targeting SPMs and other resolution mediators to promote resolution could be a promising future option in addition to current regimens (Fig. 3b), which also avoids general immune suppression and the subsequent risk of opportunistic infections. However, one of the challenges with utilising SPMs is the rapid degradation by various cell types in the inflamed tissue. Therefore, both delivery and/or increased protection against degradation have to be optimised in such therapeutic options. Therapeutic administration of PUFAs in DSS-colitis mouse models have shown to reduce pro-inflammatory cytokines and NF-κB activation^132,137,138^. Importantly, SPM analogues with improved inactivation-resistance have been synthetically produced which shall enable further investigations into their potential benefits for the treatment of chronic intestinal disorders^139^.

In conjunction to this, AS of pre-mRNA has not been extensively studied in regard to intestinal inflammatory conditions and intestinal fibrosis, despite its biological prevalence (more than 95% of multi-exonic human genes undergo AS)^140^. We^16^ and others^15,73^ have shown that AS is relevant for epithelial barriers both at homeostasis and in pathologies. Indeed, serving as a proof of concept that therapies correcting AS can work, the use of drugs which act as splice-switching oligonucleotides^141^ is currently approved for the treatment of spinal muscular atrophy^142^.

Microbiota studies in relation to human diseases are currently heavily researched, yet the current goal is to move away from correlation studies of perturbed commensal communities to particular diseases towards a functional understanding of how such changes impact intestinal health. Ultimately, the hope is to identify key molecules derived from microbes that can be used to promote resolution and barrier repair (Fig. 3b). Bacterial-derived metabolites can be produced by gut bacteria from dietary components (e.g. SCFAs which are generally anti-inflammatory), synthesised de novo (e.g. polysaccharide A which induces secretion of IL-10 from CD4^+^ T cells) or are host-derived metabolites which are biochemically modified by gut bacteria (e.g. taurine which enhances epithelial barrier function)^143^. These molecules represent yet another option for the modulation of inflammation.

Importantly, while dampening excessive uncontrolled inflammation is often necessary to treat chronic inflammatory disorders, there should also be a combined focus on the restoration of the epithelial barrier via resolution mechanisms (Fig. 3). This combined effort of alleviating disease and repairing the barrier may ultimately lead to long-lasting effects that prevent relapsing inflammatory conditions.
